# Biomimetic Mineralization in External Layer of Decalcified Fish Scale

**DOI:** 10.3390/biomimetics7030097

**Published:** 2022-07-22

**Authors:** Yanni Zhou, Yadong Chai, Kurisu Mikami, Motohiro Tagaya

**Affiliations:** 1 Department of Materials Science and Technology, Nagaoka University of Technology, Kamitomioka 1603-1, Nagaoka 940-2188, Japan; s203377@stn.nagaokaut.ac.jp (Y.Z.); yadong_chai@stn.nagaokaut.ac.jp (Y.C.); s223193@stn.nagaokaut.ac.jp (K.M.); 2 Research Fellow of the Japan Society for the Promotion of Science (DC), 5-3-1 Koji-machi, Chiyoda-ku, Tokyo 102-0083, Japan

**Keywords:** fish scales, hydroxyapatite, mineralization, simplified simulated body fluid, alkali treatment, osseous layer, collagen

## Abstract

The mineralization process of the osseous layer, which is highly calcified *in vivo*, was successfully imitated by the immersion process of the decalcified fish scales in simplified simulated body fluid (SSBF). An alkali treatment was used to modify the native collagen in the decalcified Tilapia fish scale. After the alkali treatment, the mineralization was facilitated in SSBF. The XRD patterns and SEM-EDS observation results demonstrated that the externally-mineralized layers by the immersion process were highly similar to the osseous layer containing lower-crystalline hydroxyapatite, suggesting that the simple biomimetic precipitation process was developed.

## 1. Introduction

**Scheme 1:**
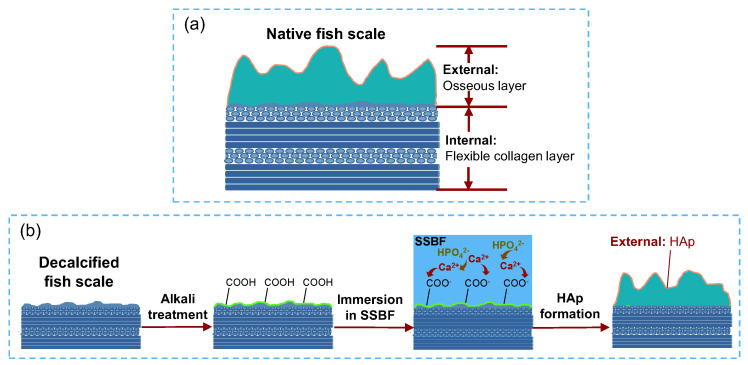
Illustration of the possible cross-sectional structures of (**a**) native fish scale and (**b**) the mineralization process of the decalcified fish scale treated with alkali and immersion in SSBF.

Fish scale has a great mechanical property as a kind of fish skin barrier, which comes from the characteristic hierarchical structures [1,2,3]. The fish scales generally have similar structures and compositions irrespective of the kind of fish [4,5]. The structures of fish scales can be mainly divided into two external and internal layers (Scheme 1a) [1,2,6,7,8]. The external layer is highly calcified by hydroxyapatite (HAp, Ca_10_(PO_4_)_6_(OH)_2_) and is called the osseous layer [6,8], which serves as the barrier against external threats [9]. The internal layer is composed of lamellar collagen fibrils, which are aligned in different directions with a lower degree of calcification [2,6]. On the other hand, bone tissue is also mainly composed of HAp and collagen fibrils, and the composite structure is very similar to that of the fish scales [2,5]. Hence, fish scale is expected to be applied for a bone regeneration field as a natural, environmental-friendly and biocompatible material [5,10,11]. Since the HAp composition affects the physicochemical properties of the bone regeneration materials [12,13,14,15], our group have researched and reported the HAp and the related biofunctional nanoparticle precipitation on the fish scales so far [16,17]. The gold nanoparticles were synthesized in the hierarchical HAp nanostructures of the natural fish scale templates [17], suggesting the confined functional and biomolecular hosts by utilizing the HAp in fish scales. Therefore, the clarification of the mineralization process of HAp in the fish scales as well as the reproduction is of vital importance for future studies of the development of bone regeneration materials as well as the bio-templated nanostructures. 

In this study, the decalcified fish scales were immersed into a simplified simulated body fluid (SSBF) [18,19,20,21], which mimics the inorganic ion concentrations and pH in biological solution to develop the characteristic mineralization process in fish scales to clarify and reproduce the mineralization process *in vitro* of the scales (Scheme 1b). For effective and controllable mineralization, an alkali treatment to the decalcified fish scales before the SSBF immersion was focused (Scheme 1b). We suggested that the alkali-treated native collagen in the fish scales will convert into atelocollagen, where the residual amide groups in asparagine and glutamine will be hydrolyzed to generate carboxy groups (Scheme S1) [22,23,24,25]. The carboxy group acts as an adsorption site for the calcium ions to promote mineralization [26,27]. Therefore, the decalcified fish scales with similar structures to the highly calcified external osseous layer were prepared, as compared with the case in the undecalcified scales.

## 2. Materials and Methods

The dried Tilapia fish scales (Shanghai, China) before (S) and after the (DS) decalcification were obtained from Japan Tuna Bait Co., Ltd. The decalcified fish scales were immersed into a NaOH aqueous solution (0.1 M) for 2 min, according to our previous study [27], and then were washed by ultrapure water and were dried to obtain the alkali-treated fish scales, which were named as DS-K. According to the previous report [28], SSBF (pH = 7.4) having 2.0-times ion concentrations (Na^+^, 284 mM; Ca^2+^, 5 mM; Cl^−^, 290 mM; HPO_4_^2−^, 2 mM; and Tris, 100 mM) was prepared and used. Both DS and DS-K were statically immersed into SSBF at 37 °C and then were washed by ultrapure water. Subsequently, the scales were dried at 37 °C for 12 h. Here, the obtained fish scales were named as DS-***X***SSBF and DS-K-***X***SSBF, where ***X*** means the immersion time in SSBF (***X*** = ***0***, ***24***, ***48***, ***72*** h).

The HAp forming ability in the fish scales was evaluated by Alizarin-red-S staining method using 29 mM of aqueous Alizarin-red-S solution, which is a common staining method and binds to calcium ion allowing the identification of the calcified areas. S, DS-***X***SSBF and DS-K-***X***SSBF were immersed in the Alizarin-red-S solution at room temperature for 30 min. The stained fish scales were then washed with ultrapure water and observed using a digital camera and optical microscopy. For the luminance extraction, the optical microscopy pictures were analyzed and deconvoluted by the RGB-separation technique, the red line profile was extracted, and the average R-brightness values were calculated. Moreover, the characterization was performed using X-ray diffraction (XRD), and desktop-type scanning electron microscope (SEM) with energy dispersive X-ray spectrometry (EDS). For Ca and P elements in the scales, the element mapping analysis was performed by K_α_ ray energies (Ca: 3.690 keV, P: 2.013 keV). 

## 3. Results and Discussion 

**Figure 1:**
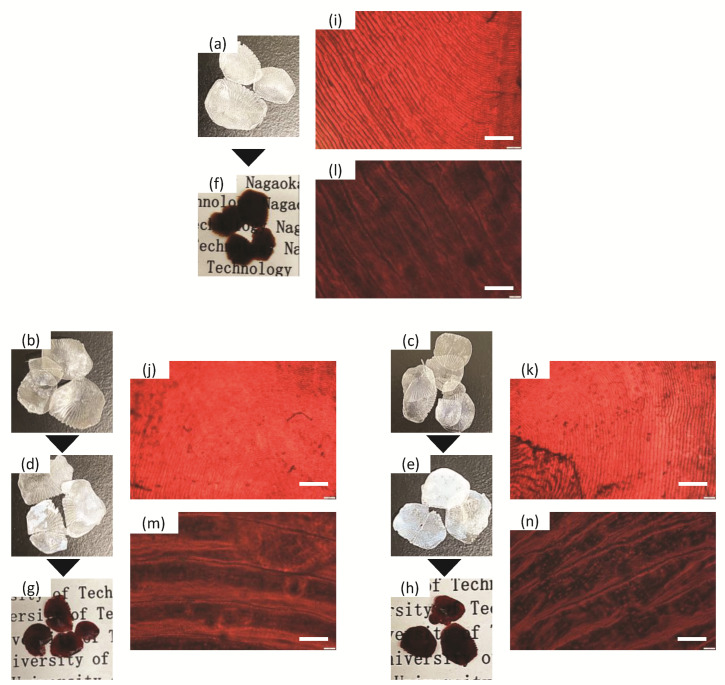
Representative photographs of (**a**) S, (**b**) DS-***0***SSBF, (**c**) DS-K-***0***SSBF, (**d**) DS-***72***SSBF, and (**e**) DS-K-***72***SSBF, and the Alizarin-red-S-stained (**f**) S, (**g**) DS-***72***SSBF and (**h**) DS-K-***72***SSBF and their optical microscope images of the stained (**i**,**l**) S, (**j**,**m**) DS-***72***SSBF and (**k**,**n**) DS-K-***72***SSBF with the scale bars of (**i**–**k**) 400 and (**l**–**n**) 40 μm.

Figure 1 shows the representative photographs and optical microscopy images of the scales before and after the immersion in SSBF and subsequent staining. S (Figure 1a) exhibits less transparency due to the presence of the osseous layer containing calcium phosphate compounds, and DS-***0***SSBF (Figure 1b) and DS-K-***0***SSBF (Figure 1c) show the transparency, indicating no calcium phosphates. After the immersion, DS-K-***72***SSBF (Figure 1e) was significantly opaquer than the case in DS-***72***SSBF (Figure 1d), suggesting that the alkali treatment promoted the calcium phosphate mineralization process. By staining with Alizarin-red-S, S, DS-***72***SSBF and DS-K-***72***SSBF turned out red (Figure 1f–h), indicating the effective mineralization. The optical microscopy images of the stained scale surfaces (Figure 1i–n) exhibited the red-colored growth rings, indicating the characteristic calcium phosphate precipitation on the osseous layers of DS-***0***SSBF and DS-K-***0***SSBF. The average R-brightness for S, DS-***72***SSBF and DS-K-***72***SSBF in the optical microscopy images was 144, 109 and 151 cd/m^2^, respectively. The value of DS-K-***72***SSBF showed a higher value than that of DS-***72***SSBF and was similar to that of S, indicating the effectiveness of the alkali treatment. 

**Figure 2:**
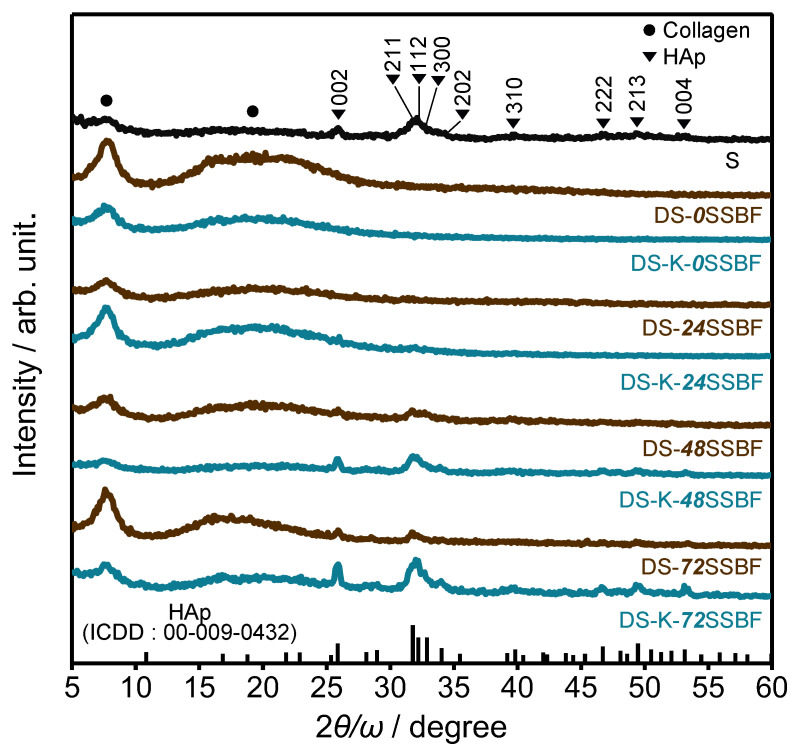
XRD patterns of the scales before and after the immersion in SSBF (incident degree (ω) of X-ray: 0.5˚).

Figure 2 shows the XRD patterns of the S, DS and DS-K immersed in SSBF for 72 h. In all the scales, there were sharp and broad diffractions at around 7.5 and 19.2˚, which corresponds to the characteristic diffractions of the collagen aggregation structures in the scales [29]. For S, the diffraction patterns attributed to the low-crystalline HAp phase were observed, whereas the HAp diffractions were not observed in DS-***0***SSBF and DS-K-***0***SSBF, indicating sufficient decalcification. After the immersion for 48 h, the diffractions due to HAp phase appeared and were intensified by the immersion for 72 h. The change with the passage of time will provide information on the nucleation and crystal growth of HAp in the scales. The DS-K-***72***SSBF exhibits higher intensity of the diffractions as compared with the case in DS-***72***SSBF, suggesting that the alkali treatment promoted the HAp formation in the decalcified fish scales. As the biomimetic synthesis, we also proposed that the crystalline state of DS-K-***48***SSBF was similar to that of S.

**Figure 3:**
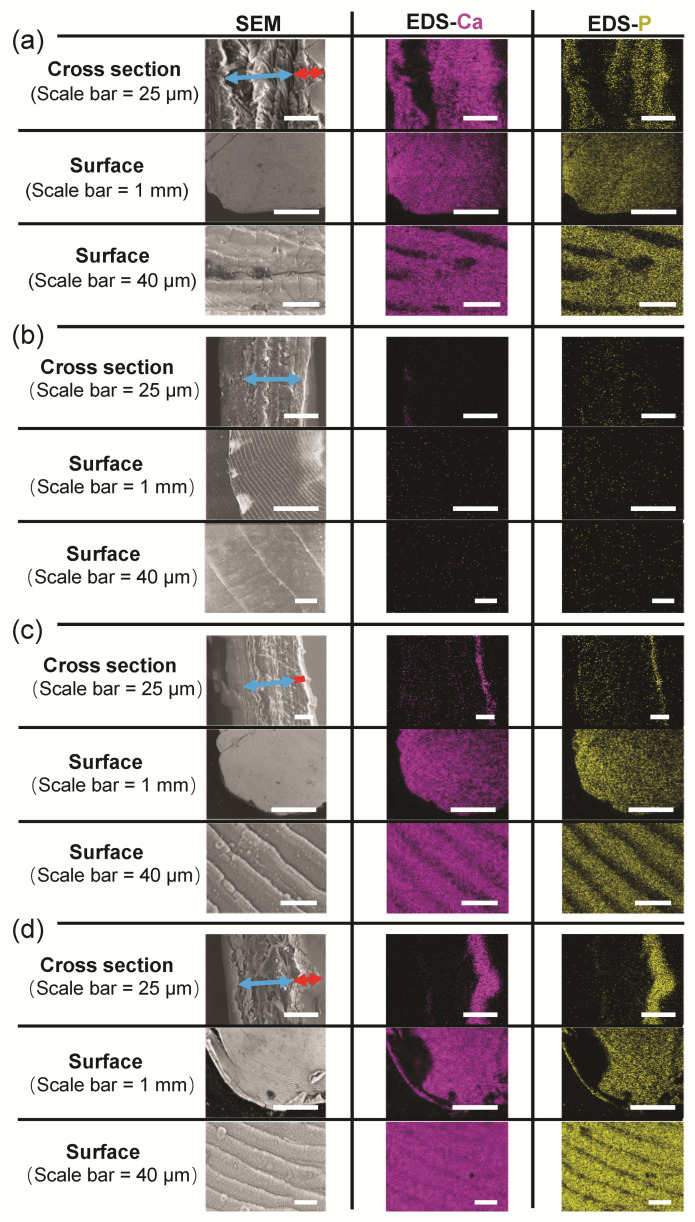
Representative SEM and EDS elemental mapping images of (**a**) S, (**b**) DS, (**c**) DS-***72***SSBF and (**d**) DS-K-***72***SSBF. The mapping was displayed with the Ca (purple-color) and P (yellow-color) dots. The blue and red arrows represent the internal and external layer regions, respectively.

Figure 3 shows the representative cross-sectional and surface SEM and EDS mapping images of the scales. In the cross-sectional SEM images of S, DS, DS-***72***SSBF and DS-K-***72***SSBF, the internal layer, which is composed of plywood structure with lamellar collagen fibrils, was observed, and the thicknesses were about 53 ± 6, 44 ± 3, 68 ± 3 and 42 ± 5 μm, respectively. From the cross-sectional EDS mapping images, the internal layer of S was calcified, and there was no internal mineralization in DS-***72***SSBF and DS-K-***72***SSBF (Figure 3c,d). From the cross-sectional SEM and EDS mapping images (Figure 3a,c,d), the external layers, which are highly calcified by HAp, were observed in S, DS-***72***SSBF and DS-K-***72***SSBF with the thicknesses of 24 ± 6, 7 ± 1, and 19 ± 5 μm respectively, whereas the external layer was not observed for DS (Figure 3b), suggesting that the immersion in SSBF could realize the mineralization of external layer. We calculated the percentage of the external layer (i.e., mineralization part) to the total thickness for S, DS-***72***SSBF and DS-K-***72***SSBF, which were about 45, 10 and 45%, respectively. The external layer thickness of DS-K-***72***SSBF was higher than that of DS-***72***SSBF, and had a similar external layer thickness ratio to the case in S, suggesting the successful modification of surface collagen by alkali treatment and the promotion of HAp precipitation. In the surface SEM images of all the scales, the characteristic growth rings could be seen. In the surface EDS mapping images of S, DS-***72***SSBF and DS-K-***72***SSBF, we could clearly observe the edges of the scales, indicating the massive and uniform precipitation of HAp on the surfaces. Meanwhile, the highly-similar growth ring structures of the scale surface to S indicate that the external mineralization processes of the calcified scales can be reproduced by the immersion in SSBF. Moreover, Table S1 shows the atomic percentages of the fish scales by the cross-sectional EDS analysis. The percentages of Ca and P increased with increasing immersion time, and the contents in DS-K-***X***SSBF were higher than that of DS-***X***SSBF. In the representative SEM and EDS elemental mapping images of DS-***24***SSBF, DS-K-***24***SSBF, DS-***48***SSBF and DS-K-***48***SSBF (Figure S1), we could see that the covered areas of Ca and P were expanded, and the mineralized external layer thicknesses increased with increasing the immersion time. Therefore, we imitated the external mineralization process of the scales, and the decalcified fish scales with similar structures to the highly calcified external osseous layers were successfully prepared through the alkali treatment process.

## 4. Conclusions

The mineralization process of the osseous layer *in vivo* was successfully imitated by the immersion in SSBF of the decalcified fish scales. An alkali treatment was used to modify the native collagen surfaces in the decalcified Tilapia fish scale. After the alkali treatment, it was found that the mineralization was facilitated in SSBF. The XRD patterns and SEM-EDS observation results demonstrated that the externally-mineralized layers by the immersion process were highly similar to the osseous layer containing lower-crystalline HAp, suggesting that the successful simple biomimetic precipitation process was developed. As a future challenge, our group will report on the internal layer mineralization of the fish scales by a biomimetic process. Moreover, the successful mineralization in SSBF suggests the possibility of supporting the biofunctional molecules or nanoparticles in the fish scale by modifying the SSBF solution. Accordingly, we can claim the great potential of the fish scales for the big interactive applications in the future.

## Data Availability

Data is available upon request from the authors.

## Supplementary Materials

The following supporting information can be downloaded at: https://www.mdpi.com/article/10.3390/biomimetics7030097/s1, Scheme S1: Illustration of the surface functional group change of the native collagen by treating with alkali; Table S1: Atomic percentages of the fish scales by the cross-sectional EDS analysis. Each value was the average taken over three different regions; Figure S1: Representative SEM and EDS elemental mapping images of DS-***24***SSBF, DS-K-***24***SSBF, DS-***48***SSBF and DS-K-***48***SSBF.
