# Supplementary material for: Biomimetic Mineralization in External Layer of Decalcified Fish Scale
Source: Biomimetics (Basel). 2022 Jul 22;7(3):97. doi: 10.3390/biomimetics7030097 (PMC9331227; doi:10.3390/biomimetics7030097)
Supplement: Supplementary file 1 [file biomimetics-07-00097-s001.zip › biomimetics-1818167-supplementary.pdf]

Electronic Supplementary Information

Biomimetic mineralization in external layer of decalcified fish scale

Yanni Zhou [a], Yadong Chai [a,b], Kurisu Mikami [a], Motohiro Tagaya [a,*]

[a] *Department of Materials Science and Technology, Nagaoka University of Technology, Kamitomioka 1603-1, Nagaoka, Niigata 940-2188, Japan.*

[b] *Research Fellow of the Japan Society for the Promotion of Science (DC), 5-3-1 Koji-machi, Chiyoda-ku, Tokyo 102-0083, Japan.*

*** Author to whom correspondence should be addressed:**

Tel: +81-258-47-9345; Fax: +81-258-47-9300, E-mail: tagaya@mst.nagaokaut.ac.jp

Scheme S1

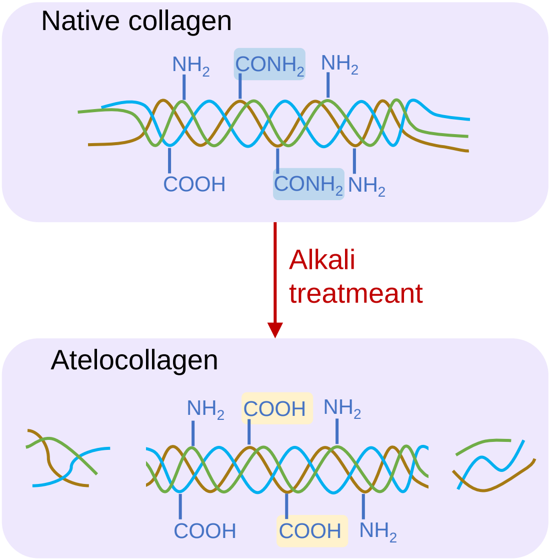

Scheme S1. An Illustration of the surface functional group change of the native collagen by treating with alkali In this case, the native collagen sometimes can be changed to atelocollagen states.

Table S1

Table S1. Atomic percentages of the fish scales by the cross-sectional EDS analysis. Each value was the average taken over three different regions.

	C	O	Ca	P	Na
S	34.1	43.6	15.41	6.57	0.28
DS-***0***SSBF	69.9	29.6	0.05	0.00	0.49
DS-K-***0***SSBF	70.1	28.9	0.02	0.01	0.89
DS-***24***SSBF	70.5	28.7	0.23	0.01	0.62
DS-K-***24***SSBF	68.4	29.0	1.57	0.80	0.21
DS-***48***SSBF	68.3	29.8	1.21	0.60	0.17
DS-K-***48***SSBF	61.9	29.4	1.75	0.60	6.35
DS-***72***SSBF	67.9	29.3	1.77	0.88	0.22
DS-K-***72***SSBF	55.1	38.6	2.52	1.27	2.55

Figure S1

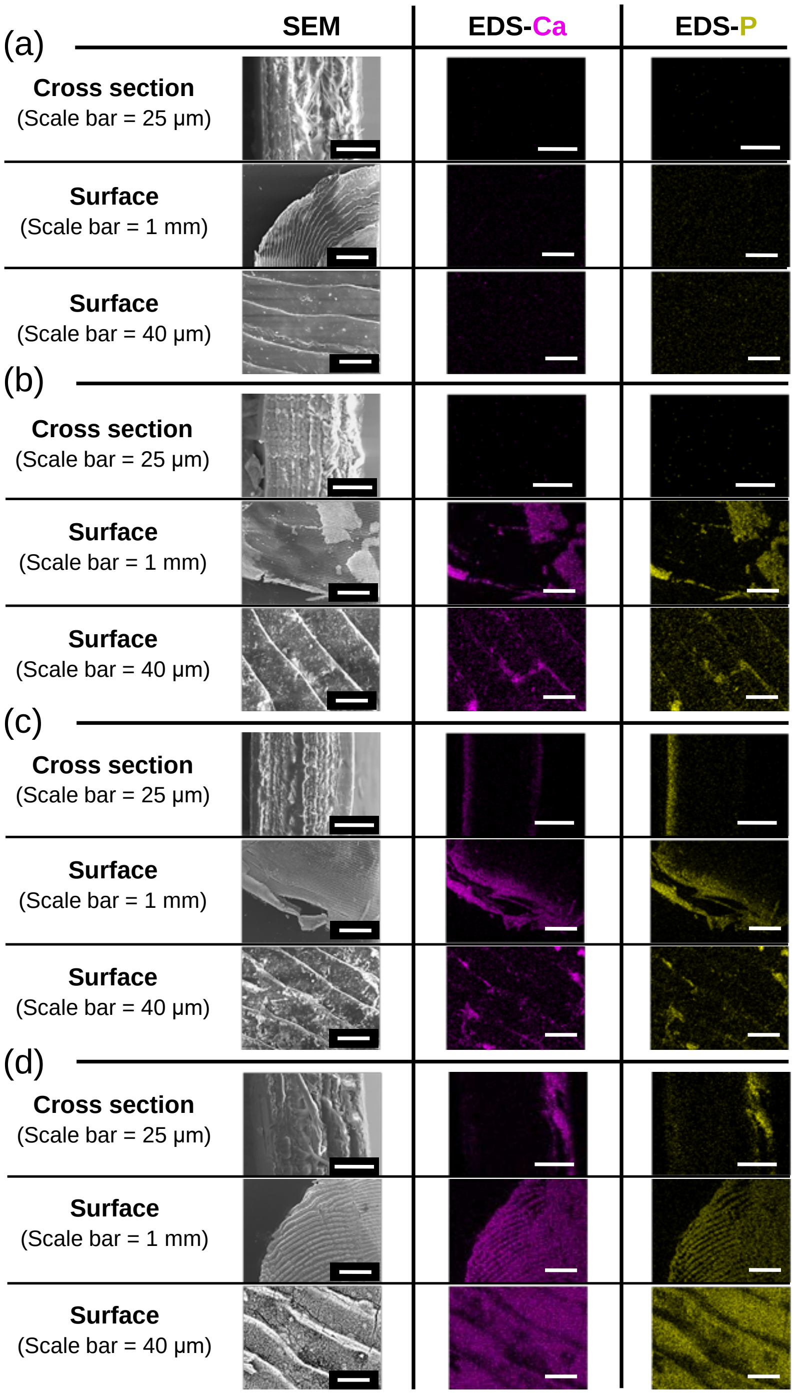

Figure S1. Representative SEM and EDS elemental mapping images of (a) DS-***24***SSBF, (b) DS-K-***24***SSBF, (c) DS-***48***SSBF, and (d) DS-K-***48***SSBF. The mapping areas of EDS-Ca and EDS-P are displayed with the Ca (purple-color) and P (yellow-color) dots.
